# Progress Toward Poliomyelitis Eradication — Worldwide, January 2021–March 2023

**DOI:** 10.15585/mmwr.mm7219a3

**Published:** 2023-05-12

**Authors:** Scarlett E. Lee, Sharon A. Greene, Cara C. Burns, Graham Tallis, Steven G. F. Wassilak, Omotayo Bolu

**Affiliations:** ^1^Epidemic Intelligence Service, CDC; ^2^Global Immunization Division, Global Health Center, CDC; ^3^Division of Viral Diseases, National Center for Immunization and Respiratory Diseases, CDC; ^4^Polio Eradication Department, World Health Organization, Geneva, Switzerland.

Since the World Health Assembly established the Global Polio Eradication Initiative (GPEI) in 1988, two of the three wild poliovirus (WPV) serotypes (types 2 and 3) have been eradicated, and global WPV cases have decreased by more than 99.9%. Afghanistan and Pakistan remain the only countries where indigenous WPV type 1 (WPV1) transmission has not been interrupted. This report summarizes progress toward global polio eradication during January 1, 2021–March 31, 2023, and updates previous reports (*1*,*2*). In 2022, Afghanistan and Pakistan reported 22 WPV1 cases, compared with five in 2021; as of May 5, 2023, a single WPV1 case was reported in Pakistan in 2023. A WPV1 case was reported on the African continent for the first time since 2016, when officials in Malawi confirmed a WPV1 case in a child with paralysis onset in November 2021; neighboring Mozambique subsequently reported eight genetically linked cases. Outbreaks of polio caused by circulating vaccine-derived polioviruses (cVDPVs) can occur when oral poliovirus vaccine (OPV) strains circulate for a prolonged time in underimmunized populations, allowing reversion to neurovirulence (*3*). A total of 859 cVDPV cases occurred during 2022, an increase of 23% from 698 cases in 2021. cVDPVs were detected in areas where poliovirus transmission had long been eliminated (including in Canada, Israel, the United Kingdom, and the United States). In addition, cocirculation of multiple poliovirus types occurred in multiple countries globally (including Democratic Republic of the Congo [DRC], Israel, Malawi, Mozambique, Republic of the Congo, and Yemen). The 2022–2026 GPEI strategic plan targeted the goal of detecting the last cases of WPV1 and cVDPV in 2023 (*4*). The current global epidemiology of poliovirus transmission makes the likelihood of meeting this target date unlikely. The detections of poliovirus (WPV1 and cVDPVs) in areas where it had been previously eliminated underscore the threat of continued poliovirus spread to any area where there is insufficient vaccination to poliovirus (*3*). Mass vaccination and surveillance should be further enhanced in areas of transmission to interrupt poliovirus transmission and to end the global threat of paralytic polio in children.

## Poliovirus Vaccination

In April 2016, trivalent OPV (tOPV), consisting of Sabin strain types 1, 2, and 3, was withdrawn from routine immunization programs and supplementary immunization activities (SIAs)* worldwide and replaced with bivalent OPV (bOPV, containing Sabin-strain types 1 and 3). Routine immunization programs worldwide provide either 3 doses of bOPV and 1–2 doses of injectable inactivated poliovirus vaccine (IPV) or IPV alone. Seroconversion after IPV vaccination protects against disease caused by all three polio serotypes but does not protect against poliovirus transmission. Because of cocirculation of cVDPV2 and other poliovirus serotypes, GPEI authorized administration of tOPV during SIAs in Afghanistan and Pakistan during 2017–2020, in Yemen during 2021–2022, and in areas of Somalia during 2022–2023. In response to cVDPV2 outbreaks, monovalent OPV Sabin type 2 (mOPV2) is approved for outbreak response use in SIAs and has been used most recently in Somalia. Because of the risks for reversion to neurovirulence associated with Sabin-strain OPV2 in areas with low immunity, the World Health Organization (WHO) granted emergency use listing of novel OPV2 (nOPV2) in November 2020 (*5*); nOPV2 is more genetically stable than the Sabin strain (*6*) and has been used in SIAs since March 2021. Challenges of nOPV2 supply during the time of this report have resulted in delayed SIAs in response to cVDPV2 outbreaks (*3*).

In 2021, the estimated global coverage with ≥3 doses of IPV or OPV (Pol3) among infants by age 1 year during routine immunization was 80%^†^; estimated coverage with 1 full dose or 2 fractional doses^§^ of IPV (IPV1) in OPV-using countries was 79%. Global coverage with Pol3 and IPV1 declined from 2019 values of 85% and 83%, respectively, when the COVID-19 pandemic severely disrupted health services. In Afghanistan, 2021 national Pol3 coverage was 71% and IPV1 coverage was 67%. Pakistan’s 2021 national coverage estimates were 83% for both Pol3 and IPV1. In Malawi, 2021 Pol3 and IPV1 coverage was 89% and 92%, respectively, and in Mozambique, 2021 coverage estimates for Pol3 and IPV1 were 67% and 70%, respectively (*7*). Immunization coverage estimates in subnational levels of these countries are often substantially lower.

During January 1, 2021–March 31, 2023, GPEI supported 48 countries in implementing 219 SIAs, during which approximately 988 million bOPV, 616,000 IPV, 960,000 fractional IPV, 90 million mOPV2, 595 million nOPV2, and 100 million tOPV doses were administered. In 2022, lot quality assurance sampling (LQAS)^¶^ surveys after SIAs indicated performance gaps in high-risk districts in Afghanistan, Malawi, Mozambique, and Pakistan (*8*–*10*).

## Poliovirus Surveillance

Poliovirus transmission is primarily detected through case-based syndromic surveillance for acute flaccid paralysis (AFP) in persons aged <15 years, with confirmation of poliovirus by testing stool specimens at one of the 144 WHO-accredited laboratories in the Global Polio Laboratory Network in 91 countries (Table 1). In 2022, AFP surveillance reviews in 34 countries at high risk for poliovirus spread indicated that 26 (76%) countries met targets for the two primary surveillance indicators at the national level.** Because of the high proportion of asymptomatic infections, environmental surveillance (ES), the systematic sampling and testing of sewage for poliovirus, can supplement AFP surveillance to detect poliovirus transmission and improve overall surveillance sensitivity. The total number of ES samples collected in countries with poliovirus transmission increased from 8,945 samples from 36 countries in 2021 to 12,259 samples from 40 countries in 2022 (Table 2).

**TABLE 1:** Number of poliovirus cases, by country — worldwide, January 1, 2021–March 31, 2023*

Country	No. of cases							
	2021		2022		Jan–Mar 2022		Jan–Mar 2023	
	WPV1	cVDPV	WPV1	cVDPV	WPV1	cVDPV	WPV1	cVDPV
**Countries with WPV1 detections (cVDPV type)**								
Afghanistan (2)	4	43	2	0	1	0	0	0
Pakistan (2)	1	8	20	0	0	0	1	0
Malawi (1)	1	0	0	4	0	0	0	0
Mozambique (1,2)	0	2	8	26	1	4	0	3
**Countries with reported cVDPV cases (cVDPV type)**								
Algeria (2)	0	0	0	3	0	0	0	0
Benin (2)	0	3	0	11	0	0	0	2
Burkina Faso (2)	0	2	0	0	0	0	0	0
Burundi (2)	0	0	0	1	0	0	0	0
Cameroon (2)	0	3	0	3	0	0	0	0
Central African Republic (2)	0	0	0	5	0	0	0	5
Chad (2)	0	0	0	44	0	5	0	5
Côte d'Ivoire (2)	0	0	0	0	0	0	0	1
Democratic Republic of the Congo (1,2)	0	28	0	504	0	58	0	31
Eritrea (2)	0	1	0	1	0	1	0	0
Ethiopia (2)	0	10	0	1	0	0	0	0
Ghana (2)	0	0	0	3	0	0	0	0
Guinea (2)	0	6	0	0	0	0	0	0
Guinea-Bissau (2)	0	3	0	0	0	0	0	0
Indonesia (2)	0	0	0	1	0	0	0	3
Israel (1,3)	0	0	0	1	0	1	0	1
Liberia (2)	0	3	0	0	0	0	0	0
Madagascar (1)	0	13	0	14	0	5	0	9
Mali (2)	0	0	0	2	0	0	0	0
Niger (2)	0	18	0	15	0	2	0	0
Nigeria (2)	0	415	0	48	0	26	0	1
Republic of the Congo (2)	0	2	0	1	0	0	0	0
Senegal (2)	0	17	0	0	0	0	0	0
Sierra Leone (2)	0	5	0	0	0	0	0	0
Somalia (2)	0	1	0	5	0	2	0	1
South Sudan (2)	0	9	0	0	0	0	0	0
Sudan (2)	0	0	0	1	0	0	0	0
Tajikistan (2)	0	35	0	0	0	0	0	0
Togo (2)	0	0	0	2	0	1	0	0
Ukraine (2)	0	2	0	0	0	0	0	0
United States (2)	0	0	0	1	0	0	0	0
Yemen (1,2)	0	69	0	162	0	83	0	0
**Total**	**6**	**698**	**30**	**859**	**2**	**188**	**1**	**62**

**Abbreviations**: cVDPV = circulating vaccine-derived poliovirus; WPV1 = wild poliovirus type 1.

* Data are current as of May 5, 2023.

**TABLE 2:** Number of circulating wild polioviruses and circulating vaccine-derived polioviruses detected through environmental surveillance — worldwide, January 1, 2021–March 31, 2023*

Country	Reporting period							
	Jan 1–Dec 31, 2021		Jan 1–Dec 31, 2022		Jan 1–Mar 30, 2022		Jan 1–Mar 30, 2023	
	No. of samples	No. of positives (%)	No. of samples	No. of positives (%)	No. of samples	No. of positives (%)	No. of samples	No. of positives (%)
**Countries with reported WPV1-positive samples (no. and % of isolates refer to WPV1)**								
Afghanistan	481	1 (0.2)	702	22 (3.1)	164	0 (—)	172	17 (9.9)
Pakistan	887	65 (7.3)	1220	37 (3.0)	275	0 (—)	362	3 (0.8)
**Countries with reported cVDPV-positive samples (cVDPV type) (no. and % of isolates refer to cVDPVs)**								
Afghanistan (2)	481	40 (8.3)	702	0 (—)	164	0 (—)	172	0 (—)
Algeria (2)	52	0 (—)	76	18 (23.7)	14	0 (—)	33	8 (24.2)
Benin (2)	143	1 (0.7)	109	8 (7.4)	36	0 (—)	39	3 (7.7)
Botswana (2)	0	0 (—)	22	4 (18.2)	0	0 (—)	25	1 (4.0)
Burkina Faso (2)	110	1 (0.9)	151	0 (—)	38	0 (—)	36	0 (—)
Burundi (2)	40	0 (—)	34	6 (17.6)	7	0 (—)	11	6 (54.5)
Canada (2)	0	0 (—)	58	2 (3.4)	0	0 (—)	12	0 (—)
Cameroon (2)	376	1 (0.3)	410	0 (—)	76	0 (—)	145	0 (—)
Central African Republic (2)	142	1 (0.7)	212	8 (3.7)	39	0 (—)	42	0 (—)
Chad (2)	64	1 (1.6)	86	5 (5.8)	14	0 (—)	14	0 (—)
China (3)	2	1 (50.0)	0	0 (—)	0	0 (—)	0	0 (—)
Côte d'Ivoire (2)	85	0 (—)	157	3 (1.9)	41	2 (4.9)	45	0 (—)
Democratic Republic of the Congo (2)	464	3 (0.6)	327	9 (2.8)	76	0 (—)	81	1 (1.2)
Djibouti (2)	71	7 (9.9)	46	12 (26.1)	10	9 (90.0)	12	0 (—)
Egypt (2)	906	12 (1.3)	645	6 (0.9)	201	4 (2.0)	139	0 (—)
The Gambia (2)	39	9 (23.1)	55	0 (—)	11	0 (—)	3	0 (—)
Ghana (2)	189	0 (—)	197	19 (9.6)	70	0 (—)	41	0 (—)
Guinea (2)	143	2 (1.4)	123	0 (—)	30	0 (—)	33	0 (—)
Iran (2)	71	1 (1.4)	68	0 (—)	17	0 (—)	10	0 (—)
Israel (2,3)	9	5 (55.6)	82	80 (97.6)	25	25 (100.0)	0	0 (—)
Kenya (2)	198	1 (0.5)	200	0 (—)	50	0 (—)	51	0 (—)
Liberia (2)	91	14 (15.4)	43	0 (—)	28	0 (—)	6	0 (—)
Madagascar (1)	393	31 (87.9)	668	96 (14.4)	158	19 (12.0)	169	24 (14.2)
Malawi (2)	0	0 (—)	353	0 (—)	54	0 (—)	56	1 (1.8)
Mauritania (2)	72	7 (9.7)	82	0 (—)	24	0 (—)	12	0 (—)
Niger (2)	204	0 (—)	301	14 (4.7)	79	2 (2.5)	66	1 (1.5)
Nigeria (2)	2,453	303 (12.4)	2218	82 (3.7)	913	46 (5.0)	294	10 (3.4)
Pakistan (2)	887	35 (3.9)	1220	0 (—)	275	0 (—)	362	0 (—)
Palestinian territories (3)	7	7 (100.0)	9	9 (100.0)	9	9 (100.0)	0	0 (—)
Senegal (2)	23	14 (60.9)	286	1 (0.3)	92	0 (—)	77	0 (—)
Republic of the Congo (2)	461	3 (1.0)	238	0 (—)	57	0 (—)	63	0 (—)
Sierra Leone (2)	214	9 (4.9)	204	0 (—)	62	0 (—)	24	0 (—)
Somalia (2)	141	1 (0.7)	231	6 (2.6)	54	1 (1.9)	67	0 (—)
Sudan (2)	103	0 (—)	160	1 (0.6)	40	0 (—)	55	0 (—)
Tajikistan (2)	27	17 (63.0)	1	0 (—)	0	0 (—)	0	0 (—)
Togo (2)	66	0 (—)	87	2 (2.3)	30	1 (3.3)	24	0 (—)
Uganda (2)	100	2 (2.0)	148	0 (—)	33	0 (—)	74	0 (—)
United Kingdom (2)	0	0 (—)	26	6 (23.1)	1	0 (—)	0	0 (—)
United States (2)	0	0 (—)	2068	47 (2.3)	81	0 (—)	952	0 (—)
Yemen (2)	37	13 (35.1)	39	25 (64.1)	15	7 (46.7)	5	0 (—)
Zambia (2)	81	0 (—)	117	3 (2.6)	27	0 (—)	40	0 (—)
**Total**	**8,945**	**608 (6.8)**	**12,259**	**531 (4.3)**	**2,951**	**125 (4.2)**	**3,290**	**75 (2.3)**

**Abbreviations:** cVDPV = circulating vaccine-derived poliovirus; WPV1 = wild poliovirus type 1.

* Data are current as of May 5, 2023.

## Reported Polio Cases and Isolations

**Countries reporting WPV cases and isolations.** In 2022, the two remaining countries with endemic WPV1 transmission, Afghanistan and Pakistan, reported two and 20 WPV1 cases, respectively (Figure) (Table 1). In Afghanistan, two cases were reported from two provinces, representing a 50% decrease from the four cases reported from two provinces in 2021 (*8*). The 20 cases reported in Pakistan in 2022 were all from security-compromised districts in Khyber Pakhtunkhwa province, representing a nineteenfold increase over the single case reported in 2021 (*9*). As of May 5, a single case of WPV1 was reported in the Khyber Pakhtunkhwa province of Pakistan in 2023. The paralysis onset dates of the latest reported WPV1 case in Afghanistan was August 29, 2022, and in Pakistan was February 20, 2023.

**FIGURE:**
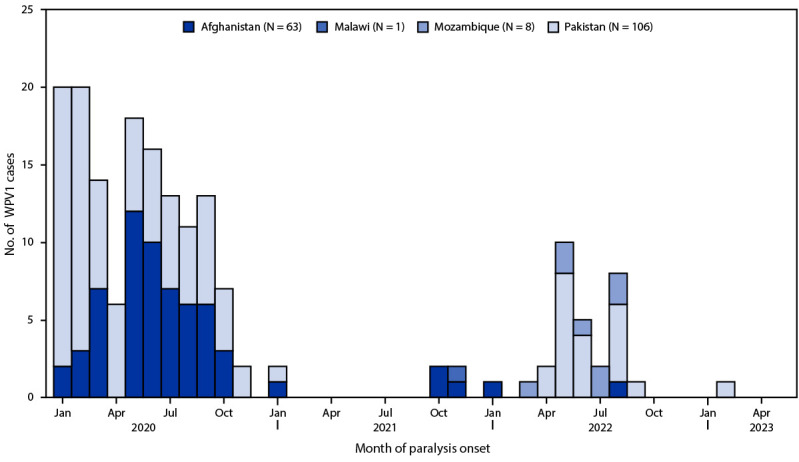
Number of wild poliovirus type 1 cases, by country and month of paralysis onset — worldwide, January 2021–March 2023* **Abbreviation:** WPV1 = wild poliovirus type 1. * Data are current as of May 5, 2023.

In Afghanistan, among 702 sewage samples collected in 2022, 22 (3%) yielded a WPV1 isolate, representing a 14-fold increase in the percentage of isolates from 0.2% (one WPV1 isolate detected in 473 samples) collected during 2021 (Table 2). In Pakistan, among 1,220 sewage samples collected during 2022, 37 (3%) WPV1-positive isolates were detected, a 57% decrease in the percentage of isolates from 7% (65 WPV1 isolates from 887 samples) in 2021. As of May 5, 2023, the latest WPV1 detections by ES were from samples taken on April 3, 2023, in Afghanistan and on February 21, 2023, in Pakistan.

In 2021, a single paralytic WPV1 case in Malawi was genetically linked to virus circulating in Pakistan and was confirmed in February 2022. In 2022, eight WPV1 cases were detected in Mozambique, genetically linked to the Malawi case, with the latest date of paralysis in August 2022 (*10*).

**Countries reporting cVDPV cases and isolations.** During January 2021–March 2023, a total of 1,619 cVDPV cases were reported from 36 countries. Six countries reported 225 cVDPV1 cases, 34 countries reported 1,393 cVDPV2 cases, and one country (Israel) reported one cVDPV3 case. DRC, Malawi, Mozambique, Republic of the Congo, and Yemen reported co-circulation of cVDPV1 and cVDPV2, and Israel reported co-circulation of cVDPV2 and cVDPV3. Global cVDPV2 cases decreased by 1.3% in 2022 (673 cases in 20 countries) compared with 2021 (682 cases in 22 countries); the 504 cVDPV cases detected in DRC represent 59% of all globally reported cVDPV cases in 2022. No cVDPV2 cases or ES detections in Afghanistan or Pakistan were reported after July 2021 (*7*,*8*). Global cVDPV1 cases increased by 1,056% (185 cases in five countries) in 2022 compared with 16 cases in two countries in 2021.

## Discussion

The 2022 increase in WPV1 cases in Pakistan’s security-challenged subdistricts of southern Khyber Pakhtunkhwa province and the ongoing circulation in contiguous districts of eastern Afghanistan form a narrow geographic band of indigenous WPV1 transmission. One major, ongoing challenge to reaching children with OPV in these reservoir districts is the substantial movement of a subpopulation at high risk between Afghanistan and Pakistan. In Afghanistan, an intensive schedule of SIAs conducted by local authorities during November 2021–September 2022 reached many previously inaccessible, unvaccinated children (*8*). However, 188,447 children residing in Afghanistan’s South Region could not be vaccinated during November 2021–September 2022 because of a regional ban on community polio SIAs. In early 2023, authorities in Afghanistan banned women from working outside the home; the ban has not substantially affected the polio program to date. In Pakistan, AFP surveillance gaps and insufficient SIA implementation quality in the areas with security issues pose substantial challenges (*9*). The successful interruption of cVDPV2 transmission in both countries in 2021 following outbreak response SIAs with tOPV and mOPV2 offers optimism that WPV1 transmission can be stopped in the near future. In 2022, both countries resumed cross-border coordination and synchronization of campaigns; intensifying and strengthening these efforts could help to mitigate cross-border WPV1 spread.

The WHO African Region detected its first WPV1 case in >5 years in 2021 in Malawi, with subsequent limited spread in Mozambique. Genomic sequence analyses for both the isolated WPV1 and cVDPV1, which cocirculated in both countries, highlight critical surveillance gaps in the region (*10*). Delays in specimen transport time, as well as some ineffective ES systems and increases in sample processing time have delayed polio detection and the subsequent response. In Mozambique, suboptimal SIA performance and decreased Pol3 coverage leave children vulnerable to further WPV1 and cVDPV transmission. Simultaneous health emergencies resulting from cholera and measles outbreaks, as well as cyclone response, in both countries have challenged the poliovirus outbreak responses. Improved SIA quality is needed to reach chronically missed children, and more sensitive surveillance will be essential in confirming the interruption of poliovirus transmission.

The 2022–2026 GPEI Strategic Plan (*4*) named the end of 2023 as the target for the last detection of both WPV1 and cVDPV2. ES detections of WPV1 transmission in Afghanistan and Pakistan and AFP detection in Pakistan in early 2023 clearly jeopardize achieving the WPV1 target. Similarly, with extensive transmission of cVDPV1 and cVDPV2 in 2023, the cVDPV detection goal is unlikely to be met by the target date. In addition, as of May 5, 2023, emergences of cVDPV2 linked to nOPV2 use had been detected in AFP cases in African countries.^††^ Although this finding was expected even with a vaccine with increased genetic stability, considering the number of doses administered, the finding indicates the need to implement high-quality response SIAs to raise immunity in all children, independent of the vaccine type used. The major hurdles to reaching the cVDPV2 GPEI goals in the near future are remaining gaps in surveillance, suboptimal SIA quality in many areas, and a highly limited nOPV2 vaccine supply, resulting in delayed campaigns for a number of countries (*5*).

The detection of cVDPV transmission in regions where poliovirus transmission has long been eliminated (e.g., genetically linked cVDPV2 in Canada, Israel, the United Kingdom, and the United States) together with the importation of WPV1 genetically related to a Pakistan strain into southeastern Africa underscore the threat of continued global poliovirus spread to any area, given global migration and travel (*3*). Further, this risk is growing because of increased postpandemic vaccine hesitancy and pandemic disruptions in immunization services, with decreased Pol3 coverage globally. Progress toward polio eradication requires continued international commitment to strengthening routine immunization, enhancing global surveillance activities, increasing SIA quality, and implementing preventive bOPV SIAs with or without IPV in areas with chronically low routine immunization coverage.

**Summary**

**What is already known about this topic?**

Endemic transmission of wild poliovirus type 1 (WPV1) continues only in Afghanistan and Pakistan.

**What is added by this report?**

In 2022, Malawi and Mozambique reported WPV1 cases linked to a Pakistan strain, the first WPV1 cases in the African region since 2016. During 2022 and 2023, Afghanistan and Pakistan reported WPV1 cases. Circulating vaccine-derived polioviruses were detected in areas of the world where poliovirus had been eliminated. Cocirculation of more than one poliovirus type occurred in multiple countries.

**What are the implications for public health practice?**

The detections of poliovirus in areas where it had been previously eliminated underscore the threat of continued poliovirus spread to any area where the population is insufficiently vaccinated against poliovirus.

## Footnotes

* SIAs are mass immunization campaigns intended to stop poliovirus circulation by immunizing every child aged <5 years with 2 OPV doses, irrespective of previous immunization status.

† 2021 is the most recent year for which data are available.

§
https://www.who.int/teams/immunization-vaccines-and-biologicals/diseases/poliomyelitis-(polio)

¶ LQAS is a rapid survey method used to evaluate the quality of vaccination coverage after SIAs in predefined areas, such as health districts (“lots”). In each lot, six clusters are selected according to probability proportional to size, then 10 houses with eligible children are randomly selected within each cluster, and vaccination status is tallied. If the number of unvaccinated children in the sample is four or more, the SIA quality is interpreted to be of lower quality and mop-up activities are recommended. Conversely, if fewer than four selected children are unvaccinated, the SIA is considered to be of high quality.

** 2022 priority countries (2022–2024 Global Polio Surveillance Action Plan priority countries): *African Region*: Angola, Benin, Burkina Faso, Cameroon, Central African Republic, Chad, Côte d’Ivoire, Democratic Republic of the Congo, Equatorial Guinea, Ethiopia, Guinea, Guinea-Bissau, Kenya, Madagascar, Malawi, Mali, Mozambique, Niger, Nigeria, South Sudan, Tanzania, Togo, Zambia, and Zimbabwe; *Eastern Mediterranean Region*: Afghanistan, Iraq, Pakistan, Somalia, Sudan, Syria, and Yemen; *South-East Asia Region*: Burma (Myanmar); *Western Pacific Region*: Papua New Guinea and Philippines.

††
https://polioeradication.org/news-post/gpei-statement-on-cvdpv2-detections-in-burundi-and-democratic-republic-of-the-congo/
